# Mapping of Tilapia Lake Virus entry pathways with inhibitors reveals dependence on dynamin activity and cholesterol but not endosomal acidification

**DOI:** 10.3389/fcell.2022.1075364

**Published:** 2022-12-16

**Authors:** Reem Abu Rass, Japhette Esther Kembou-Ringert, Rachel Zamostiano, Avi Eldar, Marcelo Ehrlich, Eran Bacharach

**Affiliations:** ^1^ The Shmunis School of Biomedicine and Cancer Research, George S. Wise Faculty of Life Sciences, Tel Aviv University, Tel Aviv-Yafo, Israel; ^2^ Department of Virology, The Kimron Veterinary Institute, Beit Dagan, Israel

**Keywords:** Tilapia Lake Virus, entry, dynamin, cholesterol, endocytosis, cytoskeleton, CRM1

## Abstract

Tilapia Lake Virus (TiLV) is an emerging virus lethal to tilapia, which threatens the global tilapia aquaculture with severe implications for food security. TiLV possesses similar features to orthomyxoviruses but is classified in the sole and the monotypic genus Tilapinevirus of the family Amnoonviridae. TiLV enveloped virions encapsidate a genome comprising ten segments of single-stranded, negative RNA. Remarkably, nine of TiLV’s ten major proteins lack sequence homology to any known viral or cellular proteins. The mode of TiLV entry into tilapia cells is not known. Following the measurement of the entry window of TiLV (∼3 h), we applied a panel of inhibitors of known regulators of endocytic functions to map the molecular requirements for TiLV entry. We identified productive entry by quantification of TiLV nucleoprotein expression and the generation of infectious particles. Inhibition of dynamin activity with dynasore or dynole, or depletion of cholesterol with methyl-β-cyclodextrin, strongly inhibited TiLV protein synthesis and infectious virion production. Moreover, inhibition of actin cytoskeleton polymerization with latrunculin A or microtubule polymerization with nocodazole within the entry window resulted in partial inhibition of TiLV infection. In contrast, inhibitors of endosomal acidification (NH_4_Cl, bafilomycin A1, or chloroquine), an inhibitor of clathrin-coated pit assembly (pitstop 2), and erlotinib—an inhibitor of the endocytic Cyclin G-associated kinase (GAK), did not affect TiLV entry. Altogether, these results suggest that TiLV enters *via* dynamin-mediated endocytosis in a cholesterol-, cytoskeleton-dependent manner, and clathrin-, pH-independent manner. Thus, despite being an orthomyxo-like virus, when compared to the prototypical orthomyxovirus (influenza A virus), TiLV shows a distinct set of requirements for entry into cells.

## Introduction

Tilapia lake virus (TiLV) is an emerging pathogen discovered in 2014 (Eyngor et al., 2014; Ferguson et al., 2014) that imposes a significant threat to the global aquaculture of tilapia—the second most important group of farmed fish worldwide (Eyngor et al., 2014; Bacharach et al., 2016; Surachetpong et al., 2020). TiLV infections affect multiple tilapia organs, including the eye, skin, kidney, and brain (Eyngor et al., 2014; Ferguson et al., 2014; Bacharach et al., 2016), and may result in high mortality rates (70%–90%) in experimentally infected fish (Eyngor et al., 2014; Tattiyapong et al., 2017; Waiyamitra et al., 2021) and farmed tilapia ((Ferguson et al., 2014; Del-Pozo et al., 2017; Surachetpong et al., 2017; Behera et al., 2018). Thus, TiLV poses a risk to the food security of millions of people (Dong et al., 2017; Surachetpong et al., 2020).

TiLV is classified as a new species (*Tilapia* tilapinevirus) under the genus Tilapinevirus, family Amnoonviridae and order Articulavirales (Adams et al., 2017). Several features indicate that TiLV is an “orthomyxo-like” virus (Bacharach et al., 2016), including an enveloped virion; a single-stranded, negative-sense, segmented RNA genome (of ten segments); similar, complementary sequences at the 5′ and 3′ non-coding termini of all TiLV segments; a stretch of three to five uridines at all of the 5′ ends of TiLV genomic RNA segments; nuclear and cytoplasmic localization of TiLV mRNA, implying a nuclear site for transcription (Eyngor et al., 2014; Bacharach et al., 2016). Each of the ten genomic segments contains one primary open reading frame (ORF); only one of which shows weak sequence homology to the influenza C virus (ICV) PB1 subunit, while the other nine ORFs lack sequence homology to known viral and cellular sequences (Bacharach et al., 2016). Recently, we identified the nucleoprotein (NP) of TiLV (encoded by Segment 4) and demonstrated that, like NPs of other orthomyxoviruses, it shuttles between the nucleus and the cytoplasm and that its nuclear export is a CRM1-dependent (Abu Rass et al., 2022).

Viruses use different endocytic routes to enter the cell and reach the endosomes, where uncoating occurs. These routes are commonly defined by the protein coat enveloping the vesicles (e.g., clathrin or caveolin), by their dependence on cholesterol or specific enzymes (e.g., dynamin), and by the involvement of the cytoskeleton (e.g., actin) (Nabi and Le, 2003; Lakadamyali et al., 2004; Yarar et al., 2005; Boucrot et al., 2006; Kaksonen et al., 2006; Lajoie and Nabi, 2007; Hirschhorn and Ehrlich, 2013; Sun and Whittaker, 2013; Yamauchi and Helenius, 2013; Johannes et al., 2015; Staring et al., 2018; Moreira et al., 2021). The difference in the usage of various endocytic routes is exemplified by the influenza A virus (IAV), which is differentially dependent on clathrin-mediated endocytosis, with dependence on the cellular context (Sieczkarski and Whittaker, 2002; Lakadamyali et al., 2003; Zhang and Whittaker, 2014; Moreira et al., 2021). At the endocytic step *per se* or after internalization of virus-containing endocytic vesicles, multiple viruses exhibit differential dependence on the structure and function of the actin and microtubule cytoskeleton. This dependency is exemplified by the effects of drugs that alter cytoskeleton dynamics, such as latrunculin A or nocodazole, on the viral infection (Lakadamyali et al., 2003; Sun and Whittaker, 2007; Cureton et al., 2010; Taylor et al., 2011; Yamauchi and Helenius, 2013; Naghavi and Walsh, 2017; Staring et al., 2018).

At the endosome, uncoating commonly depends on pH-induced conformational changes to viral fusion proteins or localized acidification-dependent proteolytic activity (or both). Moreover, additional molecular features unique to the endosome (as compared to the plasma membrane), such as the presence of intracellular co-receptors or optimal cholesterol and sphingomyelin composition, may also be required for executing viral entry (White and Helenius, 1980; Nieva et al., 1994; Carette et al., 2011; Côté et al., 2011; Wang et al., 2016; Staring et al., 2018).

Despite being an orthomyxo-like virus, the mode of TiLV entry into the cells is largely unknown. Differences from other orthomyxoviruses, including the prototypic influenza viruses and the infectious salmon anemia virus (ISAV), have been suggested, as TiLV does not hemagglutinate erythrocytes, and as ammonium chloride does not inhibit its replication (Chengula et al., 2019). Here, we employed different inhibitory treatments to dissect the cellular requirements for TiLV entry and replication in tilapia cells.

## Materials and methods

### Cell lines

The E-11 cell line is derived from the striped snakehead (Ophicephalus striatus) (Iwamoto et al., 2000), and the TmB cell line is derived from bulbus arteriosus tissue of *Tilapia* mossambica (Lewis et al., 1984). The cells were grown as previously described (Kembou Tsofack et al., 2017), in media supplemented with fetal calf serum (FCS). This medium was also used for infection experiments.

### Inhibitors

The Inhibitors’ final concentrations and sources are indicated in brackets: Dynasore hydrate (80 µm; Sigma, D7693). Dynole 34-2 (8 µm; Abcam, ab120463). Methyl-β-cyclodextrin (MβCD) (5 mm; Sigma, 332615). Erlotinib hydrochloride (10 µm; Abcam, ab141930). Pitstop 2 (30 µm; Abcam, ab120687) and its negative control (30 µm; Abcam, ab120688), which has a highly related structure to pitstop 2 and fails to inhibit endocytosis even at high concentrations (hundreds micromolar). Ammonium chloride (10mM; Merck, 1-01145-1000). Bafilomycin A1 (10 nm; Sigma, B1793). Chloroquine diphosphate (CQ) (20 µm; Sigma, C6628). Latrunculin A (0.5 μg/ml; Sigma, 428021). Nocodazole (50 ng/ml; Sigma, M1404). Leptomycin B (LMB) (45 nm; Merck, L2913).

### Determination of TiLV’s titer

TiLV’s titers in culture supernatants were determined by endpoint dilution assays and calculated as Median Tissue Culture Infectious Dose (TCID_50_) (Reed and Muench, 1938). Specifically, E-11 cells were seeded in a 96-well plate. Serial dilutions of the supernatants were used to infect ten wells (per dilution). One week post-infection, cultures were monitored for cytopathic effects (CPE) (Eyngor et al., 2014).

### Protein extraction, immunoblotting, and antibodies

Cellular pellets were vigorously resuspended in 50 μl of ice-cold RIPA lysis buffer (Merck, 20–188) supplemented with a protease inhibitor cocktail (cOmplete, Roche diagnostics GmbH, 11697498001). After 30 min incubation on ice, the lysates were centrifuged (16,000 × g; 20 min; 4°C), the cleared supernatants were transferred to fresh tubes, and protein concentrations were determined by bicinchoninic acid (BCA) method (absorbance at 570 nm), and by using the QPro-BCA Kit standard (Cyanagen, 32 PRTD1). Samples were stored at −20°C until usage. Immunoblotting and antibodies were previously described (Abu Rass et al., 2022). Densitometry of the immunoblots was quantified using the ImageJ program.

### Immunofluorescence

TmB cells (with or without the above inhibitors) were seeded on collagen-coated glass coverslips, stained, and imaged as previously described (Abu Rass et al., 2022). For quantification of the percentage of infected cells, five random images were captured at low magnification (×10). The total number of cells was determined by the 4′,6-diamidino-2-phenylindole (DAPI) signal, while infected cells were identified by NP staining (fluorescein isothiocyanate–FITC—signal).

### Quantification of nervous necrosis virus (NNV) infection

E-11 cells were treated, or not, with the indicated inhibitors for 10 min prior to NNV infection (MOI = 0.15) and for 24 h post-infection (hpi). Then, cells were harvested, total cellular RNA was extracted with TRI Reagent (Sigma, T9424), and 2 μL (∼7%) of the RNA was reverse transcribed (Quantabio, qScript flex cDNA synthesis kit, 95049-100). 2 μL (10%) of the cDNA was subjected to qPCR with a StepOnePlus real-time PCR system (Applied Biosystems), using Fast SYBR Green Master Mix (Thermo Fisher Scientific, 4385612) and primers specific for NNV coat gene (Kuo et al., 2011) or actin mRNA (Abu Rass et al., 2022); the latter was used as a reference gene. The relative NNV RNA levels were calculated by the ΔΔCT values (Livak and Schmittgen, 2001).

### Live/dead assay

Cell viability was determined using the LIVE/DEAD Fixable Red Dead Cell Stain Kit (Thermo Fisher Scientific, L34972). Briefly, cells were harvested, washed, stained, and fixed according to the manufacturer’s guidelines. R-phycoerythrin signal was detected by a FACSort apparatus (Becton Dickinson) and analyzed using FlowJo software.

### Statistical analyses

In experiments comprising a single control and multiple experimental conditions, one-way ANOVA and Dunnett’s test for multiple comparisons were applied using the GraphPad Prism software. For comparisons of one treatment and its control, a two-tailed *t*-test was performed, assuming unequal variance between groups.

## Results

### TiLV enters tilapia cells within a 3 h window

**FIGURE 1:**
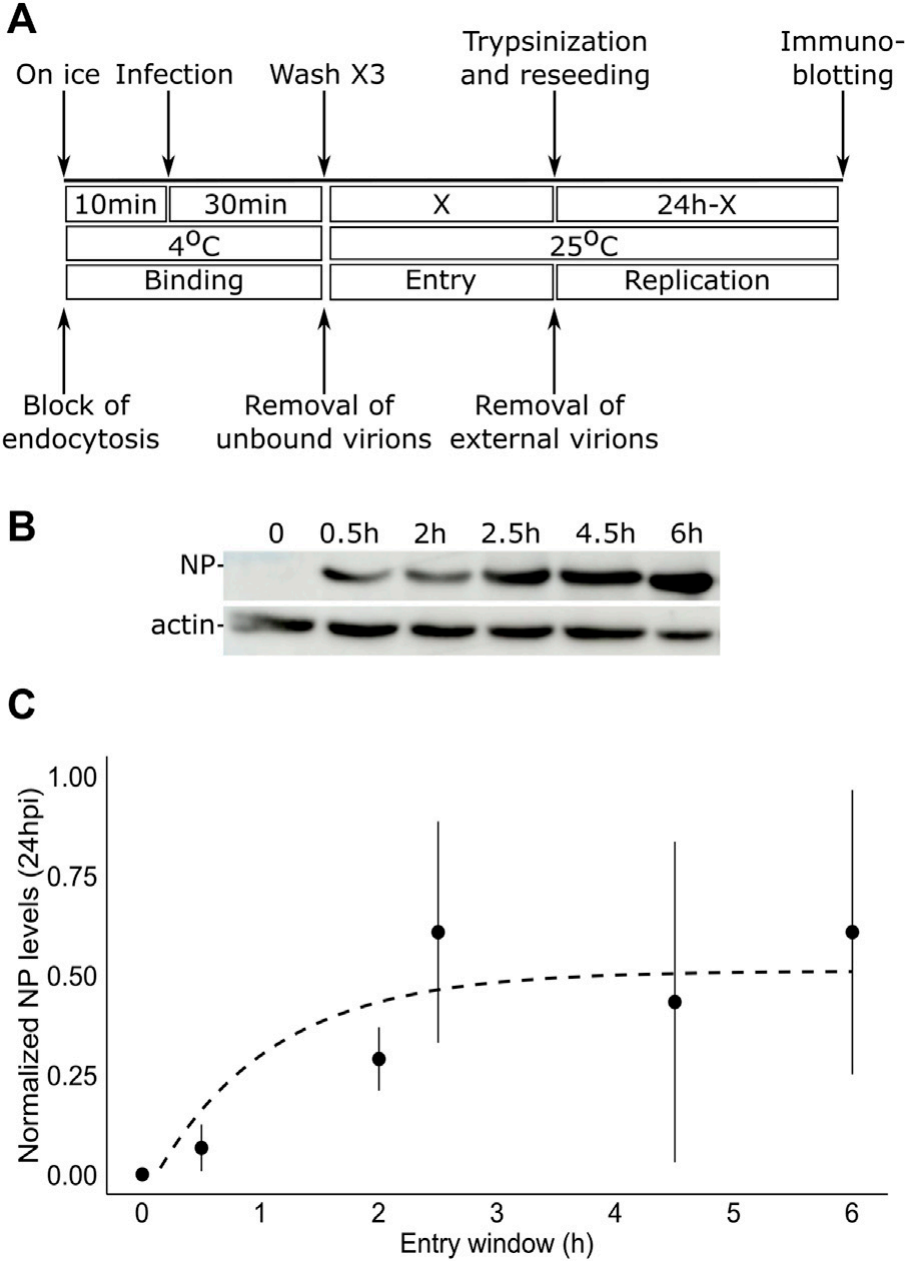
TiLV entry window in tilapia cells. **(A)** A schematic timeline and procedures used to determine the duration of TiLV entry into tilapia cells. ‘X’ represents the different times tested to allow TiLV entry. **(B)** Immunoblotting of NP and actin levels in TmB cells infected with TiLV for the indicated entry windows and extracted 24 hpi. **(C)** Quantification by densitometry of NP expression levels (normalized to actin levels) of three independent repeats of the experiment shown in **(B)**. The dashed line shows the exponential curve fitting the data, calculated by RStudio software/geom_smooth function.

To test the duration of the entry window of TiLV into tilapia cells, we designed the following protocol (Figure 1A): i) to allow binding of the virus to the cells without internalization, tilapia TmB cells (Lewis et al., 1984) were incubated on ice in the presence of TiLV particles (MOI = 1); ii) to remove unbound particles, the cells were thoroughly washed with ice-cold medium; iii) to allow entry for various periods (represented by a ‘X’, Figure 1A), we incubated the virus-bound cells with a pre-warmed medium (25°C); iv) to prevent further entry of membrane-bound particles, cells were trypsinized and reseeded. At 24 h post-infection (hpi), we harvested the cells and assessed infection levels by monitoring the expression levels of TiLV nucleoprotein (NP, aka Protein 4) (Abu Rass et al., 2022), relative to actin levels by immunoblotting (Figure 1B). At time zero (i.e., trypsinization immediately after binding; X = 0 in Figure 1A), no NP was detected, confirming the efficiency of the trypsin-mediated removal of TiLV particles. Step-wise increase in the entry window (i.e., increasing periods at 25°C prior to trypsinization) resulted in a progressive increase in the NP levels, up to about 2.5–3 h. Quantifying multiple experiments revealed a typical saturation curve with NP levels (normalized to actin levels) reaching maximal values at ∼ 3 h (Figure 1C). Our results suggest that TiLV reaches maximal cell entry at about three hpi.

### TiLV entry is dynamin-dependent

**FIGURE 2:**
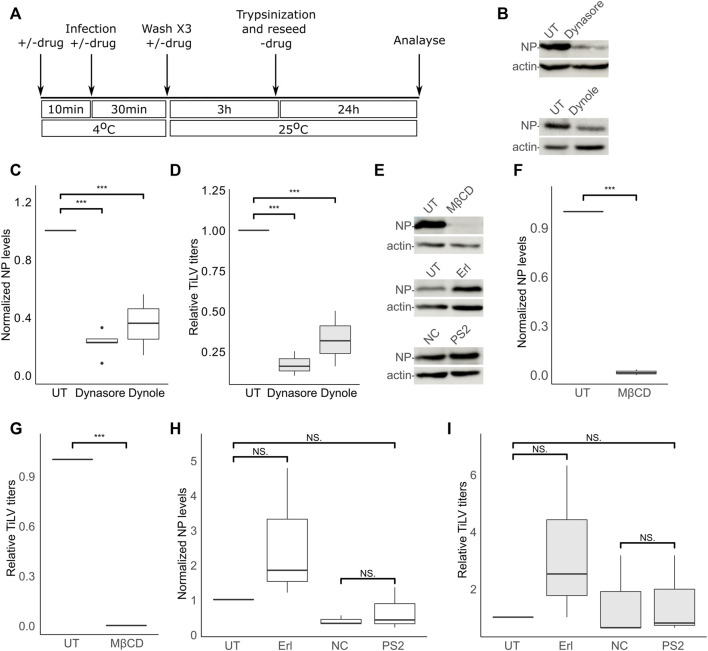
TiLV entry is dynamin- and cholesterol-dependent, and clathrin-independent. **(A)** A schematic timeline and procedures used to analyze the effects of inhibitory drugs on TiLV entry into tilapia cells. **(B–I)** Analyzes, according to **(A)**, of TiLV-infected tilapia TmB cells, untreated (UT) or treated with dynasore, dynole, MβCD, erlotinib (Erl), pitstop 2 (PS2), or the negative control (NC) of the latter. **(B,E)** Immunoblots of cellular NP and actin of representative experiments. **(C,D,F–I)** Boxplot graphs of measurements of three independent experiments for each indicated drug. **(C,F,H)** Cellular NP levels normalized to actin levels (quantified by densitometry). **(D,G,I)** Relative TiLV titers (calculated as TCID_50_/ml and shown as the ratio of the titer of the treated to untreated cultures). *, *p* ≤ 0.05; **, *p* ≤ 0.01; ***, *p* ≤ 0.001; NS, non-significant.

The dependency of TiLV entry on cellular factors is not known. To test the effect of different inhibitory drugs on TiLV entry, we modified the entry protocol described above. Specifically, the tested drugs (detailed below) were present during the binding (40 min on ice) and the entry window (3 h at 25°C). Following these two steps, the cells were trypsinized, reseeded without the drugs, and analyzed for NP expression 24 h later (Figure 2A). Additionally, supernatants were collected at 24 h post-reseeding and analyzed for the titer of infectious virions (by endpoint dilution assays). Given the central role of dynamin in clathrin-dependent and independent endocytic pathways, we first tested the effect of dynamin inhibition (with dynasore and dynole) (Macia et al., 2006; Hill et al., 2009) on TiLV entry into TmB cells. Immunoblotting of cellular NP levels in infected cells, treated or not with the drugs, revealed about 70% inhibition in treated cells (Figures 2B,C). A similar reduction was observed for the accumulation of infectious virions in the supernatants of treated cultures (Figure 2D). These results suggest that dynamin regulates TiLV entry.

### TiLV entry is cholesterol-dependent and clathrin-independent

Dynamin has been implicated in cholesterol-dependent and clathrin-dependent endocytic pathways (Nabi and Le, 2003; Macia et al., 2006; McMahon and Boucrot, 2011). To gain further insights into TiLV entry, we investigated the effects of cholesterol depletion or the effects of inhibitors known to target the clathrin pathway. To this end, we depleted cellular cholesterol with methyl-β-cyclodextrin (MβCD)—a treatment that disrupts lipid rafts and perturbs cholesterol-dependent endocytosis (Subtil et al., 1999; Nabi and Le, 2003; Ehrlich et al., 2004). The presence of MβCD within the binding and entry stages abrogated (more than 90%) TiLV infection (NP expression Figures 2E,F; infectious virion production Figure 2G), without harming cell viability (Supplementary Figure S1). To test for the involvement of the clathrin pathway, we employed a two-pronged approach: i) inhibition of the endocytic cyclin G-associated kinase (GAK) with the tyrosine kinase inhibitor erlotinib (Erl), shown to inhibit endocytosis of multiple viruses (Bekerman et al., 2017), and ii) inhibition of clathrin terminal domain function by pitstop 2 (von Kleist et al., 2011). Erl was devoid of an inhibitory effect on TiLV NP expression and titer (Figures 2H, I). To control for the activity of Erl in fish cells, we infected E-11 cells (treated or not with Erl) with the nervous necrosis virus (NNV; aka Betanodavirus)—a fish RNA virus that enters cells by clathrin-mediated endocytosis (Adachi et al., 2007; Huang et al., 2017). Erl strongly inhibited NNV infection, as evidenced by a significant reduction of cellular NNV RNA at 24 hpi (Supplementary Figure S2). For the pitstop 2 treatment, we included a negative control (NC) consisting of an inactive drug with a highly related structure to pitstop 2 (Materials and Methods). Concerning NP expression and generation of infectious particles, no significant differences were observed between cells treated with NC or pitstop 2, or between untreated cells and cells treated with pitstop 2 (Figures 2H,I). Regarding NP production, a mild reduction was observed in NC-treated, compared to untreated (UT) cells (Figure 2H), while infectious virion production was not significantly affected by either NC or pitstop 2 (Figure 2I). Together, these results suggest that dynamin activity and cholesterol are major positive factors for TiLV entry, while we find no evidence for a marked involvement of the clathrin-mediated pathway in this process. These results also suggest a dependency on endocytosis for entry.

### TiLV entry is independent of the acidification of the endosomes

**FIGURE 3:**
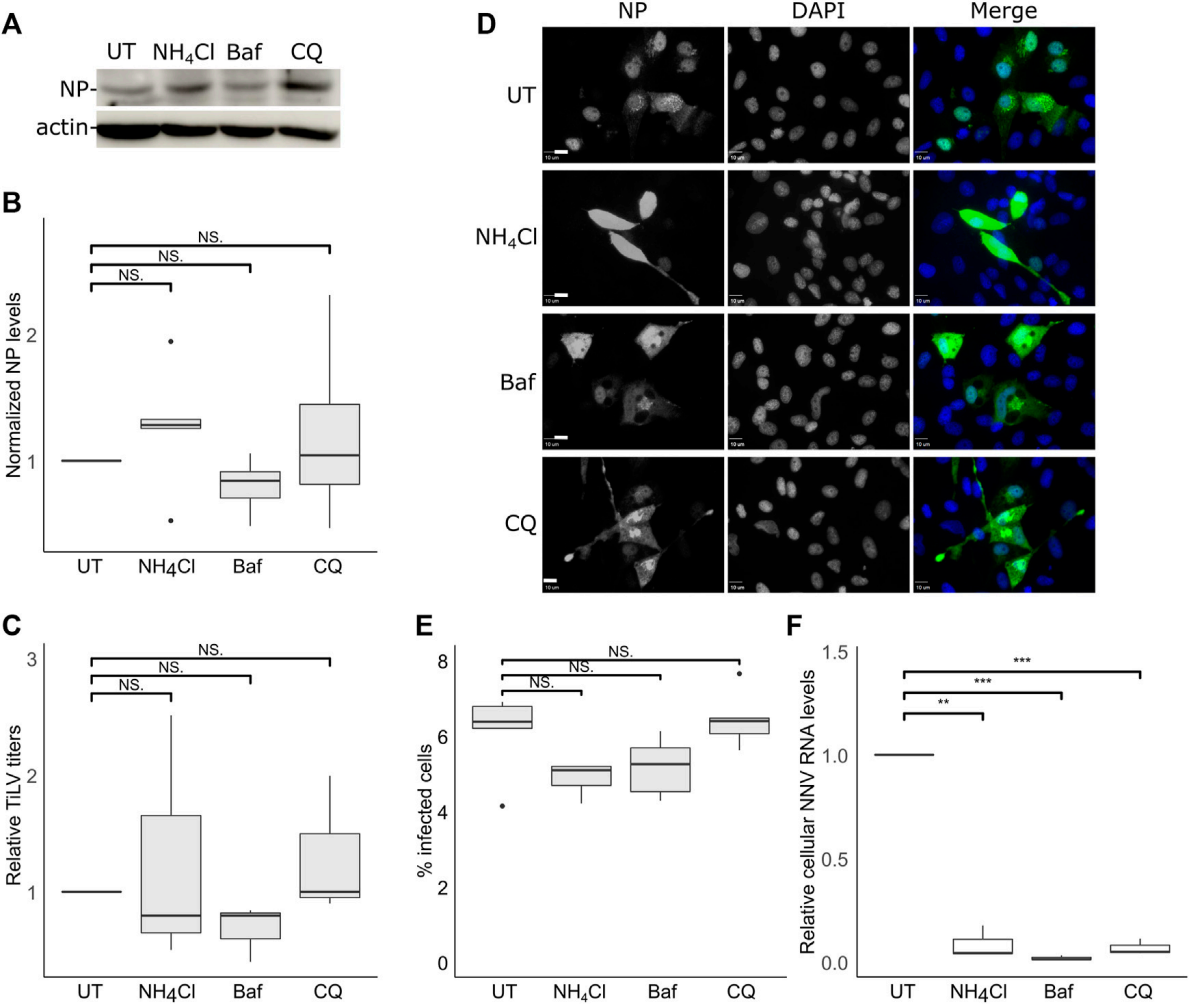
TiLV entry is independent of endosomal acidification. **(A–E)** TmB cells were treated and infected with TiLV as described in Figure 2A, with the following modification: infected cultures were incubated with the drugs for an additional 24 h after the entry window. **(A)** Immunoblotting of cellular NP and actin of a representative experiment of untreated (UT) TiLV-infected TmB cells, or treated with NH_4_Cl, Bafilomycin A1 (Baf), or chloroquine (CQ). **(B,C)** Boxplots of measurements of three independent experiments for each indicated drug. **(B)** Normalized NP levels (calculated as in Figure 2C). **(C)** Relative TiLV titers (calculated as in Figure 2D). **(D)** Immunofluorescence microscopy of TiLV-infected TmB cells at 24 h post the end of the entry window. Cells stained with anti-NP antibodies and DAPI were imaged with a confocal microscope. Bars represent 10 μm. **(E)** Cells, as described in **(D)**, were imaged at low magnification to calculate the percentage of NP-positive cells. The total number of cells was deduced from the DAPI staining. The boxplot depicts the percentage of infected cells from five image fields (∼5000 cells) per condition. **(F)** Quantification (by qRT-PCR) of cellular NNV RNA levels in infected E-11 cells. The boxplot shows measurements of three independent experiments for each indicated drug. **, *p* ≤ 0.01; ***, *p* ≤ 0.001; NS, non-significant.

The acidification and proteolysis that occur along the endocytic pathway (e.g., in endosomes) are known to license the transition from the endocytic compartments to the cytosolic milieu of the (uncoated) viral genomes. To investigate the involvement of the acidification of the endosomes in TiLV entry, we used three inhibitors of endosomal acidification: ammonium chloride (NH_4_Cl) or chloroquine (CQ)—both are lysosomotropic amines that neutralize the acidic environment; or Bafilomycin A1 (Baf), a vacuolar H + -ATPase Inhibitor. Since the acidification of the endosomes affects the viral infection after the particles enter the cells, the inhibitors were also present after the trypsinization and reseeding steps (Figure 2A) for 24 h. Immunoblotting analyzes (Figures 3A,B) did not reveal any significant reduction in NP cellular levels upon incubation with each of the three inhibitors. This result was in line with the lack of reduction in infectious virion production upon drug treatment (Figure 3C). We also tested the effect of endosomal acidification inhibitors on TiLV entry in tilapia cells by staining the infected cells (treated with the inhibitors or not) with anti-NP antibodies and imaged them by fluorescence microscopy (Figure 3D, quantified in Figure 3E). Here too, no reduction in the percentage of the infected cells (evident by NP staining) was observed upon drug treatment. As a positive control for the activity of the drugs, we infected E-11 cells with NNV, which is dependent on endosome acidification for cellular entry (Adachi et al., 2007; Huang et al., 2017). qRT-PCR measurements of cellular NNV RNA at 24 hpi revealed a potent inhibition of NNV infection by each of the three acidification inhibitors (Figure 3F), demonstrating the activity of the drugs. The lack of reduction of the NP protein levels and TiLV titers in tilapia cells is in line with the previously reported lack of reduction in viral RNA levels upon infection of non-tilapia (E-11) cells treated with NH_4_Cl (Chengula et al., 2019). Altogether, these results suggest that TiLV entry is independent of the acidification of the endosomes.

### Efficient TiLV entry is dependent on the polymerization of actin and microtubules

**FIGURE 4:**
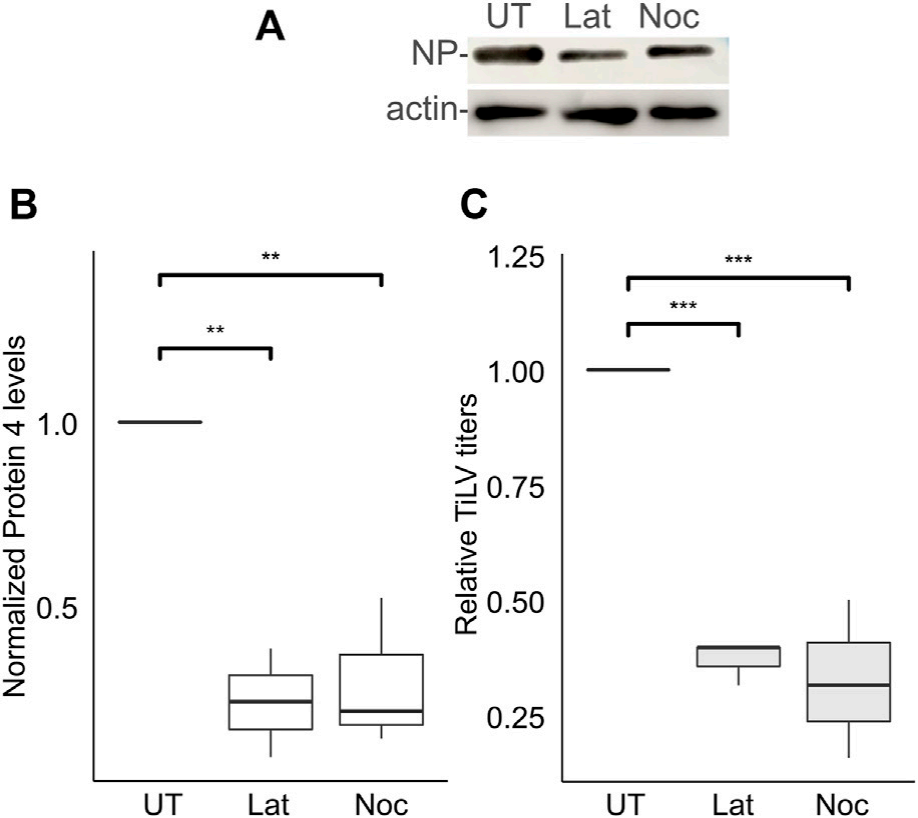
Efficient TiLV entry requires the polymerization of actin and microtubules. **(A)** Immunoblotting of cellular NP and actin of a representative experiment of untreated (UT) TiLV-infected TmB cells, or treated with latrunculin A (Lat) or nocodazole (Noc), as in Figure 2A. **(B,C)** Boxplots showing measurements of three independent experiments for each indicated drug. **(B)** Normalized NP levels (calculated as in Figure 2C). **(C)** Relative TiLV titers (calculated as in Figure 2D). *, *p* ≤ 0.05; **, *p* ≤ 0.01.

The actin and the microtubule cytoskeletons regulate distinct steps of endocytic/endosomal pathways. To address the putative involvement of actin or microtubules polymerization in the early stages of TiLV infection, we applied drugs that interfere with such polymerization - latrunculin A, or nocodazole, which target actin or microtubules cytoskeleton polymerization, respectively. To limit our analyzes to the entry window of TiLV, the drugs were administered during the binding and entry stages while later stages of infections occurred in the absence of the drugs (Figure 2A). As evident by the cellular NP levels, latrunculin A or nocodazole markedly inhibited TiLV entry (∼80% reduction; Figures 4A,B). These drugs also reduced the production of infectious TiLV particles (∼65% reduction; Figure 4C). These results suggest that efficient TiLV entry is dependent on polymerized actin and microtubule filaments.

### Inhibition of CRM1 strongly reduced TiLV titer

**FIGURE 5:**
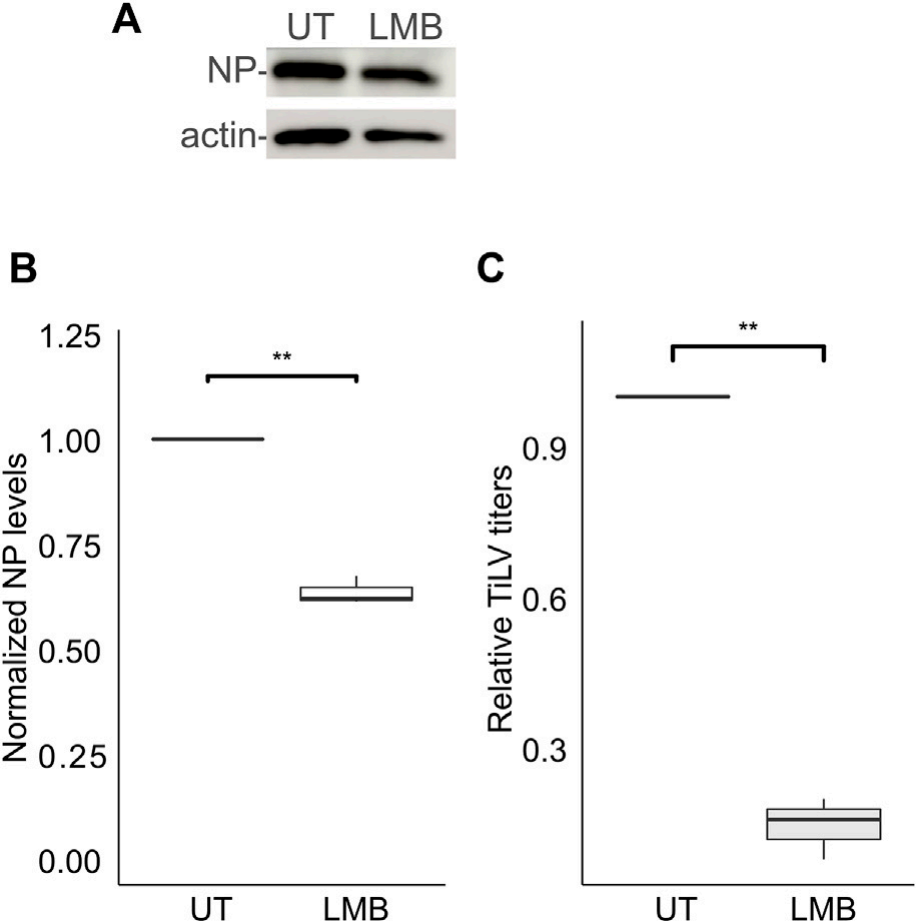
Leptomycin B strongly inhibits TiLV’s infectious virion production. **(A)** Immunoblotting of cellular NP and actin of a representative experiment of untreated (UT) TiLV-infected TmB cells, or treated with Leptomycin B (LMB). Infected cultures were incubated with the drug for an additional 24 h after the entry window. **(B,C)** Boxplots showing measurements of three independent experiments, as in **(A)**. **(B)** Normalized NP levels (calculated as in Figure 2C). **(C)** Relative TiLV titers (calculated as in Figure 2D). **, *p* ≤ 0.01.

For the vast majority of RNA viruses, the entry step culminates with the delivery of the RNA genome to cytoplasmic replication sites. However, for a subset of RNA viruses, including orthomyxoviruses, successful replication depends on the delivery of the viral RNA genome to the nucleus (Te Velthuis et al., 2021). Previously we demonstrated that TiLV RNA localizes to the cytoplasm and the nucleus (Bacharach et al., 2016), and the same is true for the NP (Abu Rass et al., 2022). Moreover, inhibition of CRM1 (aka XPO1 or Exportin-1) -dependent export by Leptomycin B resulted in further nuclear accumulation of NP (Abu Rass et al., 2022). To investigate the involvement of CRM1 activity in NP expression and the generation of infectious particles, we employed Leptomycin B to TiLV-infected TmB cells throughout all stages of infection. Leptomycin B strongly reduced TiLV titer (∼85% reduction; Figure 5C) while exerting a milder effect on the NP cellular levels (Figures 5A, B). These data complement our previous results and lend further support for the involvement of the nucleus in TiLV replication cycle and of CRM1 activity.

## Discussion

In this study, we inhibited various cellular processes related to membrane trafficking and monitored the effects of these perturbations on TiLV entry and replication in tilapia cells. These analyzes revealed that within the entry window of TiLV into tilapia cells, inhibition of dynamin, or depletion of cholesterol, strongly inhibited TiLV protein synthesis and infectious virion production. Inhibition of the cytoskeleton polymerization of actin, or microtubules, within the same timeframe also inhibited TiLV infection, although to a lower extent compared to dynamin inhibition or cholesterol depletion. In contrast, TiLV entry was not hindered by inhibitors of the endosomal acidification, clathrin-coated pit assembly, or the uncoating of the clathrin-coated vesicles. In addition, we showed that inhibition of the CRM1-dependent nuclear export strongly reduced the production of TiLV infectious particles. These results suggest that TiLV enters tilapia cells *via* dynamin-mediated endocytosis in a cholesterol-, cytoskeleton-dependent manner, and clathrin-, pH-independent manner, and the involvement of the nucleus in TiLV replication.

Many viruses, including orthomyxoviruses, enter the cells by binding to carbohydrates present on the cell surface, and such carbohydrates may be attached to protein and lipid carriers (Matrosovich et al., 2015; Maginnis, 2018). For example, sialic acid-containing glycoproteins serve as receptors for influenza viruses (Marchesi et al., 1972; Matrosovich et al., 2015), and sialic acid-containing glycolipids mediate binding and viral entry of SARS-CoV-2 (Nguyen et al., 2022). The receptor for TiLV is unknown, but while quantifying the entry window for TiLV into tilapia cells, we demonstrated that a trypsinization step efficiently removes membrane-bound TiLV particles. This fact implies that either the unknown TiLV receptor contains proteinaceous component(s), TiLV’s envelope protein bound to the receptor is trypsin-sensitive, or both.

The utilization of endocytic pathways by viruses is generally considered a mean for reaching membrane-enveloped organelles, where specific characteristics of such organelles trigger viral fusion or virally-mediated membrane disruption (Yamauchi and Helenius, 2013; Staring et al., 2018). To minimize the influence of putative off-target effects of endocytosis inhibitors on our interpretations of the observed results, we used different drugs, when applicable, to target the same pathway. Moreover, we incubated the cells with the inhibitors for only a short time (less than 4 h that spanned the entry window) and analyzed the inhibitors’ effects only after the cells were grown for an additional 24 h without the presence of the drugs. An exception to this procedure was the prolonged use of endosome acidification inhibitors, as the virus may utilize endosome acidification after virion entry to the cell. Still, none of the endosome acidification inhibitors significantly blocked TiLV infection, as opposed to the positive control (NNV infection).

Endocytic pathways are commonly defined by the protein coat that covers the incoming vesicle (e.g., clathrin or caveolin) (McMahon and Boucrot, 2011). Moreover, different pathways may also depend on common regulators—e.g., dynamin or cholesterol—shown to be involved in both clathrin-dependent and clathrin-independent pathways (Subtil et al., 1999; Nabi and Le, 2003). The dependence of TiLV entry on dynamin and membrane cholesterol strongly implies the endocytosis-mediated entry of TiLV. This notion is in line with transmission electron microscopy studies, which examined hepatocytes of TiLV-infected tilapia and located virions within membranous intracytoplasmic structures, morphologically consistent with endosomes (Del-Pozo et al., 2017). To probe for the involvement of clathrin-mediated endocytosis, we inhibited either an early step (clathrin coat polymerization, with pitstop 2) or a late step (clathrin uncoating, with erlotinib); both failed to block TiLV entry significantly. Together, these results suggest that the vesicular pathway exploited by TiLV involves dynamin and cholesterol but not clathrin. The entry of the IAV has been extensively studied, serving as a prototype for orthomyxoviruses entry (Lakadamyali et al., 2004; Sun and Whittaker, 2013). The notion that IAV enters cells *via* more than one pathway is supported by IAV sensitivity to dynasore with dependence on the lack of serum, whereas IAV enters the cells *via* macropinocytosis in the presence of serum (de Vries et al., 2011). Moreover, live cell imaging of single IAV particles demonstrated that the virus could simultaneously exploit clathrin-mediated and clathrin- and caveolin-independent endocytic pathways (Rust et al., 2004). Furthermore, genetic or chemical inhibition of clathrin, caveolin, or cholesterol-dependence pathways all failed to block IAV entry into HeLa cells (Sieczkarski and Whittaker, 2002), while in polarized Madin-Darby canine kidney type II (MDCK II) cells, IAV enters *via* clathrin-mediated endocytosis (Zhang and Whittaker, 2014). Our results suggest that TiLV shares IAV’s mode of entry in part. Specifically, while entering tilapia cells, TiLV resembles IAV in its utilization of non-clathrin carriers but differs in its dependency on dynamin activity in the presence of serum.

Actin regulates both clathrin-dependent and clathrin-independent endocytosis in a cell context-dependent manner (Yarar et al., 2005; Boucrot et al., 2006; Kaksonen et al., 2006; Lajoie and Nabi, 2007; Johannes et al., 2015); with implications to endocytosis of viruses (e.g., the vesicular stomatitis virus) (Cureton et al., 2010). Given the inhibition of TiLV entry by cholesterol depletion or latrunculin A and the lack of inhibition by drugs that perturb clathrin-mediated endocytosis, TiLV entry into tilapia cells would appear to utilize a lipid-raft/actin pathway. Notably, while latrunculin A may affect vesicle generation (an early endocytic step), the actin cytoskeleton is also involved in the intracellular transport of internalized vesicles (a post-internalization step). For example, IAV infection depends on an actin motor protein (myosin VI), which mediates vesicle motility (Sun and Whittaker, 2007). TiLV dependency on actin was weaker than its dependency on dynamin activity or cholesterol. This suggests either a partial dependency on actin for the internalization step of TiLV entry or the involvement of actin in a later step of the entry process, allowing for productive infection of internalized particles upon concomitant trypsinization and removal of latrunculin A. A partial dependency of a similar magnitude was observed concerning microtubules. Here too, inhibition of microtubule polymerization (by nocodazole, present only in the entry window) entailed a reduction in TiLV protein expression and production of infectious particles. Given that microtubules function at a post-internalization step of the endocytic pathway, our data suggest the involvement of cytoskeleton-mediated vesicle trafficking in TiLV entry. Indeed a similar scenario has been described for IAV, which depends on actin and a microtubule motor protein (dynein) to reach the endosomal compartment where viral fusion occurs (Lakadamyali et al., 2003).

A striking difference between TiLV and IAV regards their differential dependency on pH. While influenza viruses depend on endosomal acidification for their fusion with the endosomal membrane (Sun and Whittaker, 2013), TilV infection was not affected by each of the three endosomal acidification inhibitors (NH_4_Cl, bafilomycin A1, or chloroquine). In light of the pH independence of TiLV, the following open questions remain: 1. what, if any, are the unique characteristics of the endosome that triggers TiLV fusion? 2. Does TiLV benefit from such pH independence in aquatic environments? Regarding the first question, distinct viruses demonstrate dependency on endosomal components, e.g., membrane composition or the presence of additional positive factors for infection in this intracellular compartment (Staring et al., 2018). For example, the endosome-localized Niemann-pick C1 protein contributes as a co-receptor to Ebola virus infection (Carette et al., 2011; Côté et al., 2011; Wang et al., 2016), while the Semliki Forest virus requires a cholesterol and sphingomyelin membrane composition, typical of the endosome, for entry (White and Helenius, 1980; Nieva et al., 1994). The specific requirements for TiLV are yet to be identified but resemble those of Group B coxsackievirus’s entry into HeLa CCL-2 non-polarized cells. The entry of this virus depends on dynamin and cholesterol but not on either clathrin-mediated endocytosis or endosome acidification (Patel et al., 2009). Regarding the second question, TiLV and ISAV are present in aquatic environments. For ISAV, it has been suggested that it developed a molecular mechanism to counter premature rearrangements of its fusion F) protein even when exposed to acidic waters. Specifically, before receptor binding, the hemagglutinin-esterase (HE) glycoprotein of ISAV is complexed with the F protein on the surface of the virus (Eliassen et al., 2000; Fourrier et al., 2014). Structural studies on ISAV HE (Cook et al., 2017) suggested that the HE-F complex may protect the F protein from extracellular pH fluctuations (acidification) that may occur in aquatic ecosystems (Whitney, 1942; Feely et al., 2010; Shirokova et al., 2010; Ou et al., 2015). Accordingly, we speculate that the pH-independent mode of entry of TiLV may reflect TiLV’s adaptation to environmental pH fluctuations.

Following fusion, the orthomyxovirus’ RNPs reach the nucleus–a step required for their replication. In this scenario, the nuclear exit of newly synthesized RNPs is required to maintain replication and promotes assembly and egress. Indeed Leptomycin B inhibits the nuclear export of IAV NP and viral ribonucleoprotein complexes (Elton et al., 2001; Watanabe et al., 2001) and the ISAV S7ORF2 protein (Ramly et al., 2013). Furthermore, Leptomycin B Induces a nuclear accumulation of TiLV NP (Abu Rass et al., 2022) in accordance with its inhibition of the CRM1-mediated nuclear export. Here we add to this observation by showing that Leptomycin B strongly reduced TiLV titers. Notably, Leptomycin B treatment reduced NP cellular levels to a lesser extent. A possible explanation for this difference is that Leptomycin B affects the nuclear export of additional factors (cellular or viral) required for virion production.

Understanding the intricacies of TiLV entry and replication may serve as the basis for the future development of antiviral strategies against this important aquatic pathogen.

## Data Availability

The original contributions presented in the study are included in the article/Supplementary Material, further inquiries can be directed to the corresponding authors.
