# Supplementary material for: Mapping of Tilapia Lake Virus entry pathways with inhibitors reveals dependence on dynamin activity and cholesterol but not endosomal acidification
Source: Front Cell Dev Biol. 2022 Dec 16;10:1075364. doi: 10.3389/fcell.2022.1075364 (PMC9809973; doi:10.3389/fcell.2022.1075364)
Supplement: Supplementary file 1 [file DataSheet1.DOCX]

Supplementary Material

Mapping of tilapia lake virus entry pathways with inhibitors reveals dependence on dynamin activity and cholesterol but not endosomal acidification

Reem Abu Rass^1^, Japhette Esther Kembou-Ringert^1^, Rachel Zamostiano^1^, Avi Eldar^2^, Marcelo Ehrlich^1^* and Eran Bacharach^1^*

^1^Tel Aviv University, The Shmunis School of Biomedicine and Cancer Research, George S. Wise Faculty of Life Sciences, Tel Aviv, Israel

^2^The Kimron Veterinary Institute, Department of Virology, Bet Dagan, Israel

*** Correspondence:**Marcelo Ehrlich and Eran Bacharach
marceloe@tauex.tau.ac.il
eranba@tauex.tau.ac.il

# Supplementary Data

Supplementary Material should be uploaded separately on submission. Please include any supplementary data, figures and/or tables.

Supplementary material is not typeset so please ensure that all information is clearly presented, the appropriate caption is included in the file and not in the manuscript, and that the style conforms to the rest of the article.

# Supplementary Figures and Tables

For more information on Supplementary Material and for details on the different file types accepted, please see [here](https://www.frontiersin.org/guidelines/author-guidelines#supplementary-material).

## Supplementary Figures


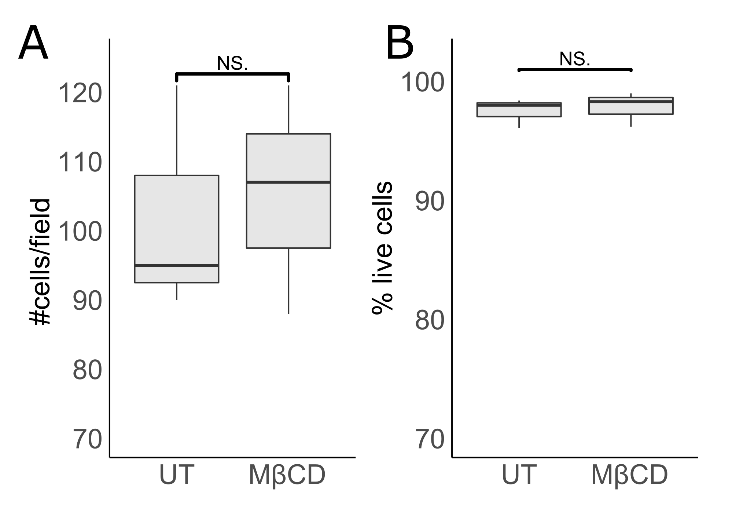


**Supplementary Figure 1.** Viability of TmB cells upon MβCD treatment. **(A)** TmB cells were treated, or not (UT), with MβCD, according to the timeline described in Figure 2A (without infection). At 24 hours post-replating, the cultures were imaged by a microscope ) EVOS Fl), and the number of cells per field was counted. **(B)** The cells from (A) were harvested, subjected to a live/dead assay, and analyzed by FACS and the FlowJo software. Boxplot depicts the percentage of live cells in each condition (n=3). NS., non-significant.


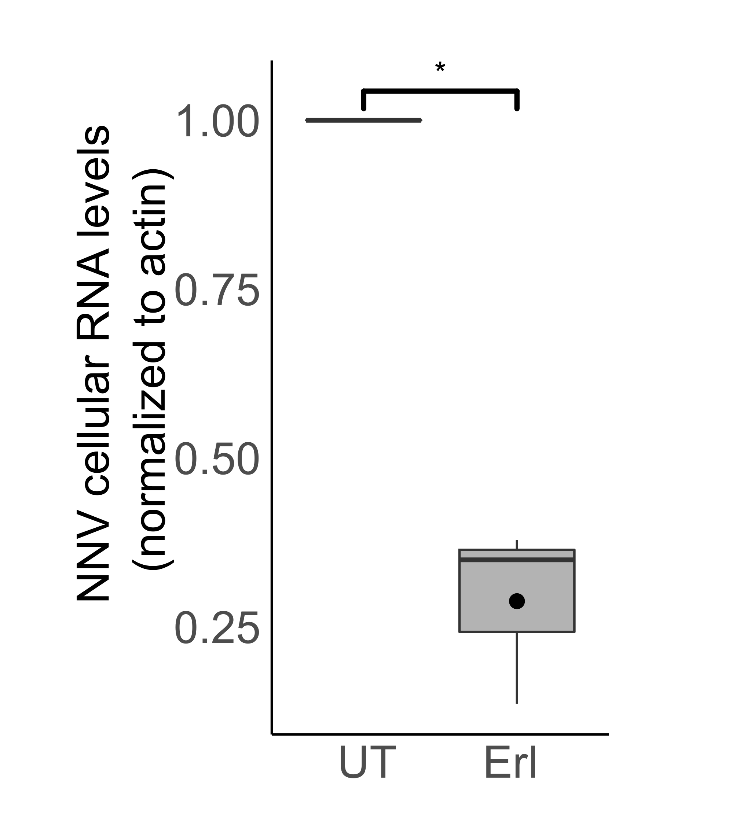


**Supplementary Figure 2.** NNV infection in E-11 cells is inhibited by erlotinib (Erl). E-11 cells were treated, or not (UT), with Erl and infected with NNV (MOI=0.15). NNV infection was quantified at 24 hpi, by qRT-PCR using total cellular RNA and primers specific for the NNV coat protein or actin. The boxplot represents relative NNV RNA cellular levels. The dot represents the mean value. *, P ≤ 0.05.
